# Differentiating superficial fungal infection from eczema using a heated dynamic-headspace skin VOC sampler: a hypothesis

**DOI:** 10.3389/fmed.2026.1856035

**Published:** 2026-06-23

**Authors:** Janmesh D. Patel, Pooja Shet, Mitchell M. McCartney, Sara Dahle, Apra Sood, Cristina E. Davis, Rivkah Isseroff

**Affiliations:** 1 VA Northern California Health Care System, Dermatology Section, Mather, CA, United States; 2 Department of Dermatology, University of California, Davis, Sacramento, CA, United States; 3 Mechanical and Aerospace Engineering, Davis, CA, United States; 4 UC Davis Lung Center, Davis, CA, United States; 5 VA Northern California Health Care Systems, Mather, CA, United States; 6 VA Northern California Health Care System, Podiatry Section, Mather, CA, United States

**Keywords:** eczema, fungi, heated dynamic headspace sampling, point of care test (POCT), skin volatile, volatile organic compound (VOC)

## Abstract

Superficial fungal infections and eczematous dermatitis frequently present with overlapping clinical features, such as erythema, scale, and pruritus, which often lead to empiric treatment and delayed diagnosis. Existing confirmatory tests, such as potassium hydroxide microscopy, fungal culture, and polymerase chain reaction, can be limited by operator dependence, turnaround time, availability, and cost. These challenges create a need for a rapid, non-invasive method to distinguish fungal infection from eczema at the point of care. We hypothesize that these conditions generate distinct skin volatile organic compound signatures because fungal lesions reflect organism-specific metabolic pathways, whereas eczematous lesions are shaped primarily by barrier dysfunction, oxidative stress, and altered microbial communities. Specifically, dermatophytes, Candida species, and Malassezia species are expected to contribute characteristic alcohols, esters, ketones, and other metabolites, while eczema is expected to be enriched in lipid peroxidation products and inflammatory barrier-related volatiles. A heated dynamic headspace sampling approach offers a practical strategy to capture these compounds by enriching semi-volatile molecules under controlled conditions while reducing interference from ambient contaminants. In parallel, advances in sorbent-based sampling, gas chromatography mass spectrometry, and portable differential mobility spectrometry support a translational pathway from biomarker discovery to clinical triage. If validated, skin volatile profiling could eventually serve as a rapid adjunctive triage approach to support confirmatory testing and guide early management decisions in selected clinical settings.

## 1 Clinical background and unmet need

Superficial fungal infections of the skin, such as dermatophyte tinea, frequently resemble eczematous dermatitis because the dominant bedside cues across these entities are similar: erythema, scaling, and pruritus, sometimes with annularity or flexural involvement. These overlaps drive diagnostic uncertainty and empiric treatment in primary and urgent care settings (1). When tinea is mistakenly treated with topical steroids as an eczematous dermatitis, treatment can temporarily suppress inflammation while allowing fungi to proliferate, producing the well-described entity “tinea incognito” and delaying appropriate therapy (1, 2). Misdiagnosis in the opposite direction, treating eczema as fungus, adds cost and drug exposure without benefit and, at a population level, contributes to antifungal-stewardship challenges amid emerging dermatophyte resistance (3).

The clinical consequences of delay are tangible. Prolonged empiric corticosteroid exposure on undiagnosed tinea worsens the extent and atypia; prolonged empiric antifungal exposure on eczematous disease wastes time and resources and may indirectly reinforce resistance pressures. Recent public-health alerts describe the spread of antifungal-resistant Trichophyton species and attribute part of the problem to the misuse and overuse of topical antifungals and corticosteroids, strengthening the case for rapid, accurate triage at first presentation (3).

Given these realities, there is a clear unmet need for a fast, non-invasive, operator-light test that can discriminate fungal infection from eczema at the point of care. In busy clinics, single-encounter tests with readouts in minutes are the norm. Many CLIA-waived antigen assays (Group A *Streptococcus*) report results at 5–10 min (4). A 15-min sampling step is feasible within typical visit workflows, but any new method should be designed to minimize hands-on time, avoid specialized credentials (or operate downstream of them), and deliver an actionable signal without sacrificing accuracy (2, 5, 6). These challenges underscore the need for rapid, reliable, and non-invasive diagnostic tools that can be deployed at the point of care.

This article is intended to advance a testable clinical and mechanistic hypothesis rather than to present a validated diagnostic method or original performance data. Specifically, we propose that superficial fungal infection and eczematous dermatitis may generate distinguishable skin volatile organic compound profiles and that heated dynamic headspace sampling provides a plausible framework for evaluating this possibility. The proposed diagnostic application remains unvalidated, and its clinical utility would require staged future study, beginning with biomarker discovery and controlled proof-of-concept testing, followed by prospective evaluation in diagnostically uncertain real-world dermatology populations.

## 2 Limitations of current diagnostic modalities

Current diagnostic approaches for suspected dermatophyte infections include potassium hydroxide (KOH) microscopy, fungal culture, and polymerase chain reaction (PCR)-based assays. KOH preparation is inexpensive and immediate, but the performance depends on sampling technique and reader experience, as well as microscope accessibility and provider time; false negatives due to inadequate specimen collection and false positives from fibers are common pitfalls (2). In tinea pedis, KOH sensitivity is approximately 73%, and specificity is approximately 42% highlight both missed cases and interpretive noise when used as a standalone test (7). Many clinics cannot perform KOH testing unless they hold a Clinical Laboratory Improvement Amendments (CLIA) Certificate for Provider-Performed Microscopy (PPM) procedures. KOH is explicitly categorized as a PPM test under CLIA, and sites without the appropriate certificate must route specimens to a certified laboratory, losing true point-of-care immediacy (5). Fungal culture improves specificity and enables speciation, but it is slow (typically requiring 2 to 4 weeks) and sensitivity is imperfect (6). Nucleic acid amplification tests (PCR) provide faster, species-level detection and can outperform conventional microscopy/culture, yet availability and adoption remain heterogeneous, and cost may present an obstacle (6, 8, 9). These limitations contribute to diagnostic uncertainty and often necessitate empiric treatment decisions. A rapid, objective, and bedside diagnostic modality would address a critical gap in dermatologic care.

## 3 Biochemical and biophysical basis for VOC discrimination

### 3.1 Biophysical basis for VOCs in skin

Skin-emitted volatile organic compounds (VOCs) arise from multiple physiological and ecological processes, including eccrine and apocrine sweat secretion, sebaceous lipids, and metabolism by resident skin microbiota; untargeted surveys and systematic reviews show hundreds of aldehydes, ketones, alcohols, and acids in the healthy “skin volatolome” (10–12). In occupied indoor environments, ozone readily reacts with unsaturated skin–surface lipids, especially squalene, to generate volatile products that include 6-methyl-5-hepten-2-one (6-MHO), geranyl-acetone, nonanal, and decanal, which can be detected within minutes and may dominate the background when ozone is present (13–15). These ozone–skin reactions are sufficiently robust that reviews now frame human skin oil as a major indoor ozone sink and source of characteristic carbonyls (15, 16).

### 3.2 VOCs in eczematous inflammation

Atopic dermatitis is characterized by a structurally and functionally impaired epidermal barrier driven by reduced filaggrin and altered stratum corneum lipids. Filaggrin insufficiency, arising from loss-of-function variants and inflammatory downregulation, weakens corneocyte cohesion and increases transepidermal water loss, establishing a “leaky” barrier prone to secondary changes in pH and microbiota (17). Ceramide composition is also perturbed: short-chain ceramides rise, and long-chain species fall, disrupting lamellar organization and correlating with diminished barrier function in patients with atopic eczema (18). Type-2 cytokine signaling further remodels the lipidome of both lesional and non-lesional skin, linking immune activation to barrier lipid abnormalities (19). Consistent with these structural defects, oxidative stress markers accumulate in the outer epidermis; studies demonstrate increased reactive oxygen species-associated damage in the stratum corneum of individuals with more severe disease and elevated epidermal oxidant load (20, 21). Lipid peroxidation of sebum and membrane lipids generates reactive aldehydes and related carbonyls, and several skin headspace markers, such as longer chain alkanals, can be traced to this chemistry (e.g., undecanal from peroxidation of specific sebum fatty acids) (22). Together, these mechanisms predict that atopic dermatitis lesions will emit a volatilome enriched in lipid-peroxidation-derived aldehydes and ketones relative to non-lesional skin. Exogenous exposures can amplify barrier failure: *in vitro* and skin-equivalent experiments show that toluene downregulates filaggrin via extracellular signal-regulated kinase (ERK), and signal transducer and activator of transcription 3 (STAT3) pathways, suggesting a plausible route by which indoor volatile organic compounds reshape both barrier biology and the overlying volatile organic compound profile (23). Observational work also associates environmental volatile organic compound mixtures with atopic dermatitis phenotypes, reinforcing the need to record exposure context when interpreting lesion headspace (24).

### 3.3 VOCs in dermatophyte metabolism

Dermatophytes are specialized for growth in keratinized tissues and secrete a suite of keratinolytic enzymes that depolymerize hair, nail, and stratum corneum proteins into assimilable peptides and amino acids, enabling trophic access to the superficial epidermis (25, 26). In culture, these taxa generate reproducible, species-distinct volatile organic compound (VOC) fingerprints; recent comprehensive gas chromatography studies across multiple dermatophyte species showed that alcohols and ketones are prominent features and that VOC spectra can differentiate taxa at species, and even strain, level resolution (27). Although sulfur-containing volatiles are well documented across fungi and sometimes accompany amino acid catabolism, their diagnostic contribution in dermatophytes remains to be defined, underscoring the need for targeted measurements in skin-relevant conditions (28). In contrast, Candida species consistently emit the aromatic alcohol 2-phenylethanol and related esters through the Ehrlich pathway, wherein phenylalanine is transaminated to phenylpyruvate, decarboxylated to phenylacetaldehyde, and reduced to 2-phenylethanol; experimental studies in *Candida albicans* link yields to growth conditions, and secretome analyses show 2-phenylethanol as a dominant volatile across *Candida* spp. (29–31). Lipid-dependent *Malassezia* species, which are common on human skin and overrepresented in sebaceous niches, rely on exogenous fatty acids and produce headspace profiles enriched in fatty alcohols and small branched alcohols when grown on lipid-containing media, offering a mechanistic rationale for VOC differences on oily skin sites or in eczematous states where *Malassezia* abundance is increased (32, 33). Together, these trophic programs predict that dermatophyte lesions will favor keratin catabolism-linked alcohols/ketones, *Candida* lesions will be marked by Ehrlich products, such as 2-phenylethanol and its acetate, and *Malassezia*-rich sites will contribute lipid-derived alcohols. Prospective, *in vivo* lesion headspace studies that resolve profiles by species and track changes during antifungal therapy are still sparse and remain a key research need to translate these laboratory patterns into actionable clinical panels.

### 3.4 Microbiome contrasts in eczema and superficial fungal infections

Eczema and superficial fungal infections differ not only in host pathology but also in the structure of their cutaneous microbial communities, which is expected to shape the volatile organic compound (VOC) milieu above lesions. In atopic dermatitis, *Staphylococcus aureus* colonization is common and often increases during flares; longitudinal pediatric studies show dominance of *S. aureus* with reduced bacterial diversity at exacerbation and partial restoration after therapy, and a meta-analysis confirms high carriage across body sites in atopic dermatitis compared with controls (34, 35). The fungal compartment likewise shifts: while healthy skin mycobiomes are typically *Malassezia*-dominated, recent studies in moderate-to-severe atopic dermatitis report increased fungal diversity, decreased *Malassezia* colonization, and greater recovery of non-*Malassezia* yeasts such as *Candida*, patterns that may vary with disease severity and site (36–38). By contrast, superficial mycoses are usually pathogen-dominated ecosystems. In tinea pedis, internal transcribed spacer-based profiling identifies *Trichophyton* as the principal fungal taxon within lesions, with accompanying shifts in the local bacterial community; in cutaneous candidiasis, *Candida* spp. often overgrow at occluded or macerated sites and can outcompete commensals (39, 40). Since microbes emit species and pathway-specific volatiles, these differing community structures plausibly yield distinct VOC blends*: S. aureus* and other bacteria release characteristic short-chain acids, alcohols, and sulfur compounds *in vitro*, dermatophytes produce species-skewed alcohols and ketones, and *Candida* spp. generate aromatic alcohols such as 2-phenylethanol via the Ehrlich pathway (27, 31, 41). These lines of evidence motivate lesion-level volatilomics stratified by organism, while highlighting the need to quantify rates of bacterial co-infection in eczema and to map how mixed communities modulate headspace profiles *in vivo*. In practice, these contrasts may be blurred by lesions that contain both inflammatory and infectious components. Eczematous lesions may be secondarily colonized by bacteria or fungi, and fungal lesions may be altered by prior corticosteroid exposure or present in inflamed skin, which could shift the observed volatile profile away from a simple host versus pathogen distinction.

### 3.5 Expected diagnostic contrast

The anticipated diagnostic contrast between eczema and superficial fungal infection arises from fundamentally different sources of volatile organic compound production. In eczema, volatile signatures are expected to reflect host barrier failure, altered epidermal lipid composition, oxidative stress, and secondary shifts in the skin microbiome. Reduced filaggrin, disrupted ceramide architecture, and type 2 inflammatory remodeling promote lipid peroxidation and accumulation of reactive carbonyl species, which together are predicted to enrich lesion headspace in aldehydes and ketones, particularly longer chain alkanals and related oxidation products. This pattern is likely further shaped by the frequent presence of *Staphylococcus aureus* and a more heterogeneous fungal community, both of which can contribute additional low molecular weight acids, alcohols, and sulfur-containing compounds.

By contrast, fungal lesions are expected to generate volatile profiles that are more directly tied to organism-specific metabolism and trophic behavior. Dermatophytes colonizing keratinized tissue should favor alcohols and ketones associated with keratin degradation and amino acid utilization, while *Candida* species are expected to contribute Ehrlich pathway products, such as 2-phenylethanol and related esters. *Malassezia*-rich environments may add lipid-derived fatty alcohols and small branched alcohols, particularly at sebaceous sites.

Taken together, these contrasts suggest that eczema will tend to show a broader host and inflammation-weighted oxidative signature, whereas fungal infection will more often display a pathogen-weighted metabolic signature with more specific alcohol, ester, and ketone features. The most clinically useful classifier will likely depend not on a single compound, but on a panel that captures the balance between host-derived oxidation products, organism-specific metabolites, and the microbial community context of each lesion.

## 4 Prior work in VOC-based diagnostics

Background VOCs can overlap with disease-associated signals; therefore; many protocols recommend parallel ambient and device blanks (or filtered make-up air) to quantify and manage environmental contributions during skin-VOC sampling (11, 12, 42). At the same time, practice varies. In breathomics and related volatilome workflows, simple ambient subtraction does not always improve discrimination and can even degrade it, likely because subtraction ignores gas-exchange and matrix effects. Several studies therefore advocate modeling or controlled sampling rather than blanket subtraction (43–45). In dermatology clinics, potential background sources extend beyond ambient room air and may include disinfectants, clothing, footwear, topical products, and patient hygiene exposures. These factors reinforce the need for structured metadata capture and may favor modeling or controlled sampling approaches rather than simple background subtraction alone.

A range of methods has been described for capturing skin VOC emissions, and review articles published in 2014 and updated in 2021 provide comprehensive summaries of reported volatiles from human skin and related body sources (10, 12). In practice, most approaches share two overarching requirements. The first is to isolate and concentrate skin emissions from the surrounding environment. The second is to collect the captured VOCs in a form that can be analyzed with sufficient chemical specificity.

To isolate emissions, investigators typically seal an open container or chamber against the skin surface, such as a glass jar or a patch-like device (46), and apply it to common sampling sites, including the forearm, chest, back, or neck. The chamber volume can then be purged with filtered air to lower the background and improve recovery of endogenous VOCs. Captured VOCs are most often concentrated on sorbent materials that absorb or adsorb analytes depending on their surface chemistry. Solid phase microextraction (SPME) fibers may be inserted into the chamber and exposed directly to the headspace, while sorbent-packed tubes can draw air across the material using a pump. Sampling durations vary, but typically range from a few to 30 min, and one study identified 15 min as an efficient duration for untargeted recovery in a heated dynamic headspace system (46). Once candidate biomarkers are established, targeted methods can reduce the need for longer sampling and analysis times, and may improve analytical measurement and diagnostic performance for a specific clinical question.

Collected VOCs are commonly analyzed offline using gas chromatography mass spectrometry (GC MS), which remains the prevailing benchmark for compound identification and quantification (10, 12). In contrast, online analytical approaches such as proton transfer reaction mass spectrometry (PTR MS) can enable real-time monitoring of skin emissions. For example, Abu Bakar and colleagues developed a handheld probe that transferred VOCs directly to an online mass spectrometer and demonstrated the approach on the armpits, forearms, and foreheads of nine volunteers (47). Taken together, these studies support the technical feasibility of collecting and analyzing skin VOCs, but they do not yet establish clinical discrimination between superficial fungal infection and eczema.

## 5 Proposed technology: heated dynamic headspace VOC sampling

### 5.1 Design rationale

We propose heated dynamic headspace sampling as a practical approach for collecting volatile organic compounds directly from the skin surface. The long-term purpose of this technology is to support the development of a rapid point-of-care adjunctive triage approach for skin lesions, including wounds, that may help identify cases in which superficial fungal infection warrants further confirmatory evaluation. If a concerning VOC pattern was detected in future validated workflows, it could prompt standard clinical testing, such as microscopy, culture, or molecular diagnostics, rather than serving as a stand-alone diagnostic result.

The rationale for heated dynamic headspace sampling is that incoming filtered air can be gently warmed within a sealed chamber, which increases recovery of less volatile compounds and can improve detection limits. Dynamic airflow also allows continuous collection of skin emissions and enrichment of analytes onto a sorbent, improving sensitivity compared with passive sampling approaches. In the published device evaluation by Davis and colleagues, experimental optimization supported a sampling time of 15 min and the selection of Tenax TA as a sorbent for untargeted recovery (46).

### 5.2 Advantages for dermatologic application

**Figure 1:**
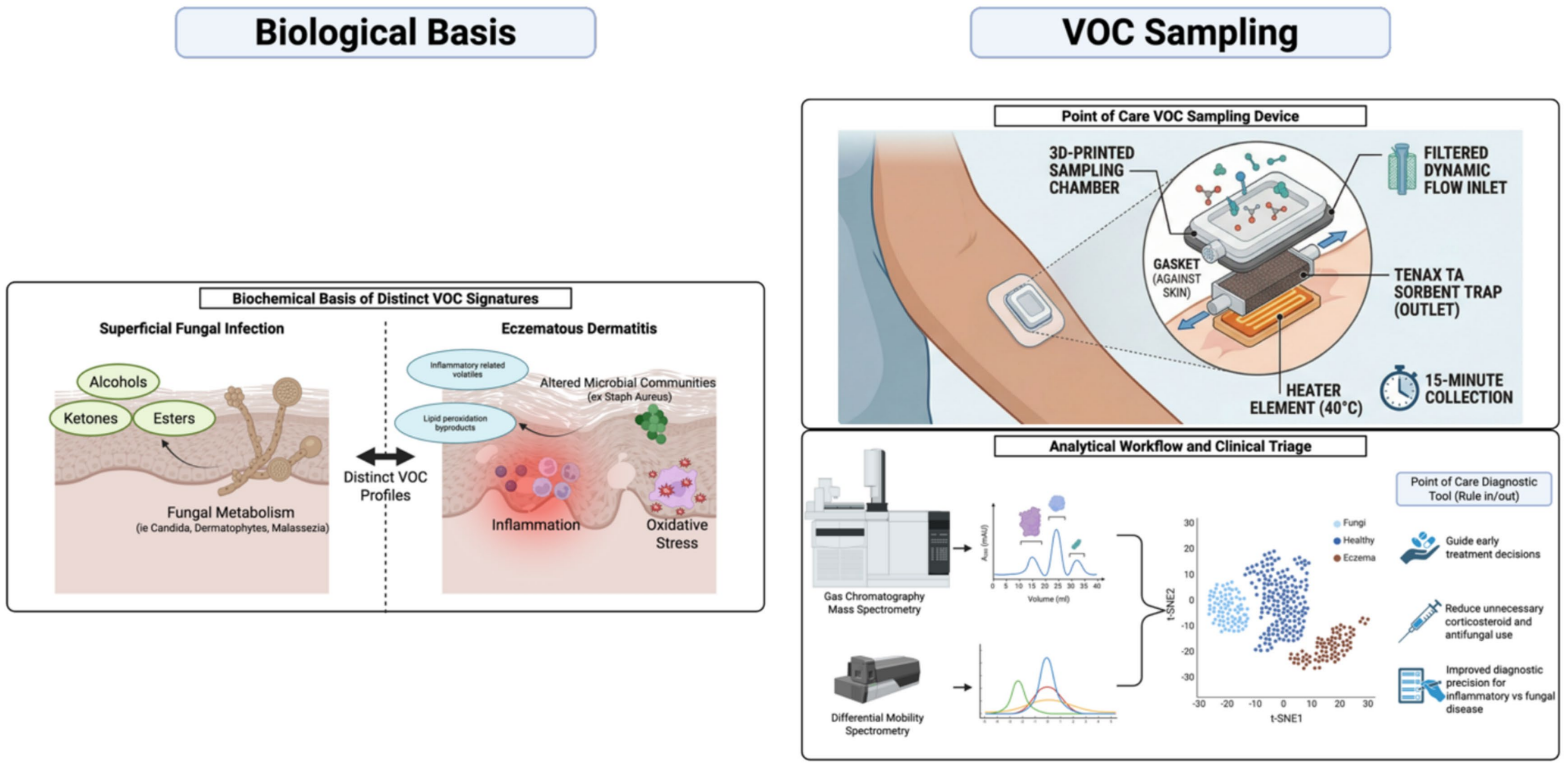
Proposed workflow for volatile organic compound (VOC)-based differentiation of superficial fungal infection and eczema. A heated dynamic headspace skin patch collects lesion VOCs under controlled airflow using a Tenax TA sorbent trap. Distinct biochemical drivers are hypothesized to generate different VOC signatures, with fungal lesions reflecting organismal metabolism and eczematous lesions reflecting inflammation, oxidative stress, barrier dysfunction, and altered microbial communities. Captured VOCs are analyzed by gas chromatography mass spectrometry and, in future translational study, potentially by portable sensing platforms. Computational classification could then be evaluated as an adjunctive triage aid to support decisions about confirmatory testing in selected clinical settings. This image was made with Biorender.

This approach offers several advantages for dermatologic use. It is non-invasive and painless, can be performed at the bedside, and is designed to support clinically practical sampling times. By improving recovery of low-abundance and less volatile compounds, heated dynamic headspace sampling may increase the likelihood of detecting diagnostically useful signals from complex skin lesions (Figure 1).

**Figure 2:**
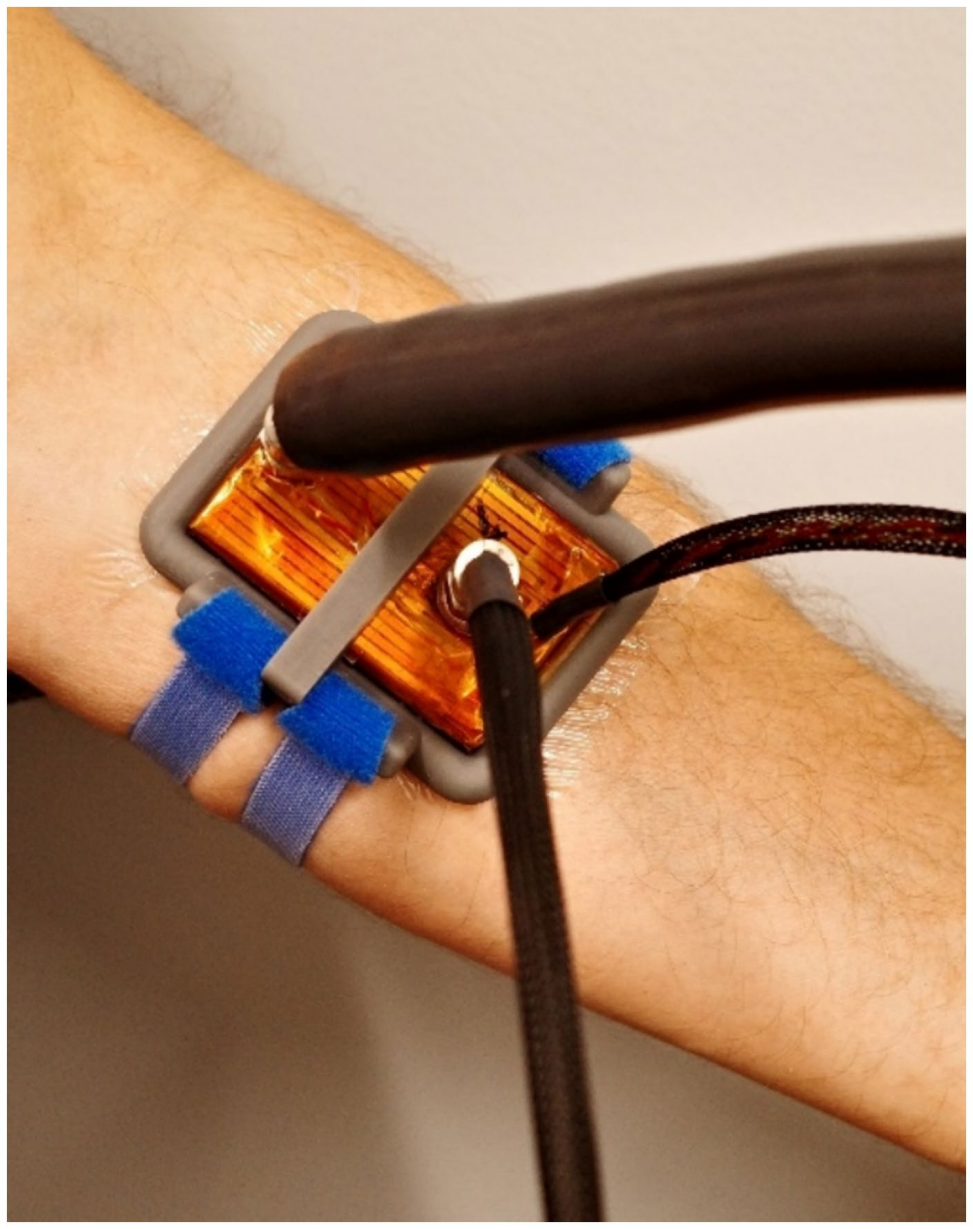
Special patch device (46) to collect skin VOC emissions, which uses a gentle warming feature to help recover less volatile species. Emissions are pumped to an attached differential mobility spectrometer (49), not shown. This device was funded by an NIH grant.

An example of a model sensor for collecting skin volatile organic compound emissions has been developed by Davis and colleagues (Figure 2) (46). The device uses a three-dimensional printed chamber that seals against the skin using a gasket and forearm straps. Incoming filtered air is gently warmed to heat the chamber, while internal temperature control is maintained at 40 °C, a threshold consistent with human safety (46). The heated headspace patch described above is also designed to support ease of use, minimal operator dependence, and patient comfort, although additional work is needed to refine form factor and workflow so that hands-on time is minimized and clinical throughput is preserved.

### 5.3 Potential for point-of-care integration

A central translational challenge is that many existing approaches are difficult to deploy at scale in clinical studies aimed at developing skin VOC diagnostics. Although mass spectrometry remains the analytical benchmark, its size, cost, and operational complexity, such as reliance on benchtop instrumentation and trained personnel, currently limit routine bedside use. In contrast, portable technologies such as ion mobility spectrometry have demonstrated potential to discriminate skin VOC profiles, supporting the concept of in-clinic screening with near-real-time data generation (48). At the point of care, VOCs can be measured using differential mobility spectrometry (DMS) devices, such as open source implementations that are microfabricated in compact formats suitable for clinical deployment (49). Differential mobility spectrometry has a history of field applications, such as defense-oriented detection of hazardous chemicals, and it operates at ambient temperatures and pressures. Selectivity can be improved by coupling DMS with gas chromatography (GC DMS) (50). Open source and semi-automated analysis tools have been developed to visualize these data and support multivariate methods, such as partial least squares discriminant analysis and random forest classification, to distinguish chemical profiles between cohorts (51–55).

Together, these features support the longer-term possibility of a point-of-care adjunctive screening platform for dermatology clinics and other ambulatory settings, contingent on substantial additional analytical and clinical validation. No Food and Drug Administration-approved skin volatile organic compound diagnostic tests currently exist. Early engagement with regulators will therefore be important to define expectations for analytical performance, reproducibility, and diagnostic accuracy. As larger datasets of human skin volatile organic compound emissions become available, these efforts can help clarify appropriate regulatory pathways and support broader implementation.

Several practical barriers would need to be addressed before routine clinical use. Sampling protocols would need to standardize lesion selection, chamber placement, temperature, flow rate, collection time, sorbent handling, device cleaning, and blank collection. Reproducibility would need to be demonstrated across operators, clinic rooms, anatomical sites, disease severities, and environmental backgrounds. The cost of the sampling device, sorbent materials, detector platform, maintenance, calibration, staff training, and data interpretation workflow would also need to be evaluated against the cost and turnaround time of existing diagnostics such as potassium hydroxide microscopy, culture, and polymerase chain reaction. A portable prototype is already being used across two clinical sites, providing a practical foundation for future assessment of reproducibility, cost, and integration into routine clinical workflows. A compact, non-invasive sampling platform may ultimately prove compatible with outpatient practice, analogous in broad concept to other adjunctive bedside assessments used in ambulatory care. However, its actual feasibility, staffing requirements, throughput, and workflow impact would need to be established prospectively.

The translational pathway for skin VOC diagnostics is likely to proceed in stages. Gas chromatography mass spectrometry would first serve as the discovery platform for compound identification and biomarker selection. A reduced panel of candidate VOCs could then be evaluated using portable technologies, such as differential mobility spectrometry or gas chromatography differential mobility spectrometry. These portable systems would require separate analytical and clinical validation before they could be considered for routine dermatologic use.

## 6 Hypotheses

As a testable hypothesis for future study, we propose that a volatile organic compound panel collected with a heated dynamic headspace skin patch at 40 °C for 15 min onto Tenax TA may enable future differentiation of superficial fungal infection from eczematous dermatitis in a point-of-care-oriented triage framework, with performance benchmarks of at least 90% sensitivity and 85% specificity, selected to reflect the current diagnostic landscape while balancing efficiency and timing requirements for point of care use. These values should be interpreted as future goals rather than expectations supported by current fungal versus eczema VOC data. This prediction is based on the expectation that fungal lesions will be enriched in organism-derived alcohols, esters, and fatty acid catabolites, whereas eczema will show more lipid peroxidation-derived aldehydes and ketones, and that heated collection with Tenax TA will improve recovery of these diagnostically useful compounds while background contaminants are accounted for through blanks and preprocessing. We did not perform preliminary validation for this hypothesis, and direct clinical comparison between eczema and superficial fungal infection remains limited, as this is an acknowledged clinical gap in the literature.

## 7 Proposed validation strategy

### 7.1 Study design

To evaluate this hypothesis, we propose a prospective case–control study comparing patients with confirmed superficial fungal infection and patients with clinically, and when available, histologically diagnosed eczema. The fungal cohort would ideally be classified using a prespecified composite reference standard that incorporates potassium hydroxide preparation, fungal culture, polymerase chain reaction, and clinical adjudication, rather than relying on a single test. Culture will serve as the primary fungal reference standard, with KOH and PCR reported as supportive or exploratory comparators. The study could also be structured such that subjects may act as their own controls to account for variability in VOC signatures among patients. This design would allow direct comparison of volatile profiles across clinically relevant groups while reflecting the diagnostic uncertainty encountered in routine practice.

An initial case–control design would be appropriate for biomarker discovery because it maximizes contrast between well-characterized fungal and eczematous lesions. However, because this approach may overestimate diagnostic accuracy relative to routine practice, a subsequent prospective study should enroll patients with clinically ambiguous scaly or pruritic eruptions before the final diagnosis is established.

### 7.2 Sampling methodology

Volatile sampling should be performed under standardized conditions using the heated dynamic headspace device at a fixed temperature, flow rate, and collection time. Skin site selection should be documented carefully, and analyses should account for anatomical variation by stratifying or modeling sebaceous, moist intertriginous, and dry sites separately when needed. Recent exposure to consumer products, topical medications, and solvents should be recorded, room air and device blanks should be collected with each session, and environmental conditions should be kept as constant as possible to reduce background variability. Lesion VOCs may be affected by patient behavior and clinic environment; therefore, validation studies should include structured metadata collection for recent use of topical antifungals, corticosteroids, emollients, antiseptics, soaps, perfumes, deodorants, sunscreens, solvents, and other hygiene products. When feasible, participants should avoid non-essential topical products before sampling, although this may not be practical during the first encounter for point-of-care use. Prior to sample collection, the area of the VOC device placement will be cleaned with ethanol. Room air and device blanks should be collected at each session, and analytes associated with ambient contamination or product exposure should be included as covariates or assessed in sensitivity analyses.

### 7.3 Analytical technique

Gas chromatography mass spectrometry should be used initially to identify candidate volatile compounds and define the chemical features most relevant for fungal versus eczematous lesions. Classification can then be built using supervised and unsupervised approaches, such as partial least squares, support vector machines, neural networks, and clustering methods, with performance assessed in calibration and validation sets. Consistent with prior volatilomic studies, discrimination will likely depend on a multicomponent signature rather than a single biomarker, and complementary analytical platforms such as gas chromatography differential mobility spectrometry may support future point-of-care translation.

### 7.4 Statistical analysis

GC-DMS datasets will be analyzed using both supervised and unsupervised machine learning approaches, such as neural networks, support vector machines, and *K*-means clustering, to identify volatile organic compound (VOC) patterns predictive of superficial fungal infection versus eczema. Multivariate regression techniques such as partial least squares (PLS) will be applied to GC-DMS-derived VOC profiles to model infection status and differentiate fungal involvement from non-infected eczematous skin. Model performance will be evaluated based on accuracy, specificity, and selectivity for classifying superficial fungal infection by splitting the data randomly into calibration and validation test sets.

Consistent with prior skin VOC investigations, multivariate approaches will rely on constellations of VOC features (10–20 compounds) rather than single biomarkers to characterize disease states. Techniques such as PLS rank chemical features according to their importance in discriminating infection status, and these chemicals will be identified using complementary mass spectrometry analysis.

### 7.5 Outcome measures

The primary outcome would be the sensitivity and specificity of volatile organic compound-based classification for differentiating superficial fungal infection from eczema. Secondary outcomes should include reproducibility across patients, robustness across anatomical sites, and stability of classification after accounting for environmental and treatment-related confounders. Additional analyses should examine mixed inflammatory infectious lesions, recently treated lesions, and steroid-modified tinea separately when sufficient sample size is available because these may represent major sources of VOC heterogeneity and reduced classifier performance.

### 7.6 Limitation

This hypothesis has several important limitations. First, it is conceptual and does not include preliminary clinical data demonstrating that volatile organic compound profiles can reliably distinguish superficial fungal infection from eczematous dermatitis in patients. Second, lesion VOC signatures are likely to be influenced by substantial biologic and environmental variability, including anatomical site, recent topical treatments, hygiene products, mixed microbial communities, and background contaminants in the clinic setting. Third, the proposed diagnostic contrast may be less distinct in real-world presentations, such as steroid-modified tinea, partially treated eruptions, or lesions with overlapping inflammatory and infectious features. Finally, although heated dynamic headspace sampling and downstream analytical platforms are technically promising, additional work is needed to establish reproducibility, define the optimal diagnostic classifier, and determine whether this approach can be translated into a practical point-of-care workflow.

## 8 Conclusion

Superficial fungal infection and eczematous dermatitis remain challenging to distinguish at the point of care because of overlapping clinical features and limitations of current diagnostic methods. The biochemical, microbial, and biophysical differences outlined in this hypothesis support the possibility that these conditions generate distinguishable skin volatile organic compound signatures. In particular, eczema is expected to reflect barrier dysfunction, oxidative stress, and mixed microbial dysbiosis, whereas fungal lesions are more likely to show organism-driven metabolic products linked to keratin utilization, lipid metabolism, and species-specific trophism. Heated dynamic headspace sampling offers a plausible method to enrich these diagnostically relevant compounds under controlled conditions while remaining compatible with downstream analytical and portable sensing platforms. If validated in prospective clinical studies, this approach could provide a rapid, non-invasive adjunct for lesion triage, improve diagnostic precision, and reduce unnecessary or inappropriate treatment in inflammatory and infectious skin disease.

## Data Availability

The original contributions presented in the study are included in the article/supplementary material, further inquiries can be directed to the corresponding author.
